# Effects of Animated Movies on the Aggression and Behavior Performance of Primary School Students and Their Control Using a Cognitive Behavioral Anger-Control Training (CBACT) Program

**DOI:** 10.3390/bs13080659

**Published:** 2023-08-06

**Authors:** Ponam Saba, Haiwen Qi, Atif Saleem, I-Jun Chen, Fahd Naveed Kausar, Muhammad Farhan Iqbal

**Affiliations:** 1 School of Education, Soochow University, Suzhou 215123, China; hwqi2019@stu.suda.edu.cn (H.Q.); 20177218002@stu.suda.edu.cn (F.N.K.); 2 School of Education, Huaibei Normal University, Huaibei 235000, China; ad668@nenu.edu.cn; 3 School of Education, Minhaj University Lahore, Lahore 54700, Pakistan; 4 Department of Education, Qurtuba University of Science and Information Technology, Dera Ismail Khan 29050, Pakistan

**Keywords:** aggression, animation, students, CBACT, behavior management

## Abstract

This work is a combination of two studies, *Study I* and *Study II*, which aimed to examine the impact of animated movies on the aggression and behavioral performance of primary school students, as well as their control using the CBACT program. In *Study I*, the influence of animated movies on 300 students from ten primary schools in Pakistan with gender differences (50% male), family systems, and viewing time duration was examined. This study was performed on 7- to 9-year-old children on four subscales of the Buss–Perry aggression questionnaire (BPAQ), three subscales of the child behavior questionnaire (CBQ), and toy selection. Following that, in *Study II* the CBACT intervention was applied to aggressive students (n = 46) selected from *Study I*. Students were randomly divided into CBACT treatment (50%) and control groups. The results of *Study I* indicated that violent animated movies had a greater impact on the aggressive behavior of male students than on that of females, while girls were more affected by watching nonviolent movies. Furthermore, male viewers from nuclear families and females from joint families showed more aggressive problems. It was also observed that aggression in students significantly increased (*p* < 0.001) with increasing viewing animation time duration (10 to 30 min). The results of *Study II* showed that aggression in the treatment group was significantly reduced (*p* = 0.000) with the CBACT program but remained constant for the control group. The findings of *Study I* showed that violent animation is strongly linked with aggression and behavioral performance in primary school students. The CBACT program in Study II indicates that students may not be completely eliminated from watching violent movies, but their aggression levels were reduced when they watched animated movies. Therefore, the CBACT program opens a new window into behavioral problem treatment, which is casually influenced by violent media.

## 1. Introduction

From a social-psychological point of view, aggression is a ‘deliberate act’ carried out by humans that intend to harm them or others who do not deserve it [1]. Humans, according to Aristotle, are social animals that interact with one another and mediate their interpersonal interactions through some factors such as feelings and emotions. This interaction sometimes hurts others physically, verbally, and mentally, leading to some of the worst effects on children, adolescents, families, and the community [2]. Therefore, aggression is considered an antisocial behavior and is considered aggressive behavior, which is explained by several theories, especially social learning theory (SLT) [3,4,5]. Moreover, such behavior becomes alarming for parents, teachers, researchers, and policymakers to control and/or reduce anger, aggression, etc., in children who need some intervention or therapy to achieve positive results [6,7,8]. Often, the media contributes its role to children’s behavior because children have a substantial attraction toward video games and animated movies, such as cartoons [9]. Many children prefer to watch their favorite characters in animated movies instead of playing outdoor games, and these characters can be violent or nonviolent.

A large body of literature demonstrates that media violence activates negative feelings and thoughts, which leads to behavioral problems in children [10,11,12]. Other researchers performed experimental studies on children in which violent and nonviolent games were reflected as animation-like characters while playing different games [13,14]. However, some experimental studies and meta-analyses have also shown the positive or negligible impact of violent media on the aggression of youth, suggesting no significant relationship between media violence and aggressive behavior [15,16,17,18]. Considering the positive and negative impact of media, it will be interesting to design a strategy that shows the influence of violent and nonviolent animated movies on the aggression and aggressive behavior of primary school students. Furthermore, the influence of watching violent animation on aggression and behavioral problems in children regarding gender differences, family systems with gender differences, and spending time duration have rarely been examined [19,20]. Additionally, several types of interventions, including child-focused, school-based, family-centered, and multiple-component programs have been previously performed in controlling and/or reducing anger and anger-related problems of animated viewers and video gamers [6,8,9]. Among these therapies, cognitive behavioral therapy (CBT) is considered more problem-focused therapy and has been previously applied to control or reduce anger-related problems in children [6,7]. It is regarded as the best school-therapy; however, parents’ involvement in lower-grade students can be helpful for the accomplishment of therapy.

Therefore, CBT and/or its forms are needed to reduce the aggression of primary school students. In this study, the effects of violent and nonviolent animations on the aggressive behavior of primary school students and their control using the CBACT intervention were investigated.

## 2. Literature Review

### 2.1. Animation Violence and Gender Differences in Aggression

Children’s perceptions of gender differences have significant attraction toward animation, and these animations can be violent, nonviolent, comedic, or non-comedic. However, the child prefers to watch his favorite character in these animations, which may lead to aggression in both genders, as many of the animations today have violent content. Previously published studies showed that children respond differently to animation, with male characters appearing more frequently (75%) than female characters (25%), demonstrating the direct, indirect, relational, and social aggression of gender differences [11,21,22,23]. Both genders have different animation selections due to different emotions, notions, and opinions. Research has shown that when viewing violent media, males show more aggression than females [22]. Others have demonstrated that males are more interested in violent animation scenes, while females generally prefer romantic and emotional characters [23]. Generally, the number of male characters in animated movies is greater than that of females due to the attractive and leading role of males during threatening situations [23]. Compared to males, the impact of animated movies violence has been less explored in females [24]. Therefore, in this study, the effect of animations was deeply examined and compared in both sexes.

### 2.2. Animation Violence in Students from Different Family Systems

Family systems and the interaction between family members play considerable roles in increasing or controlling the anger and aggression of children [5,25]. Children in Pakistan, such as those in other Asian nations, are greatly impacted by animated films and are raised in both family systems (nuclear or single and joint or extended families) as well as single-parent nuclear families (a variant of a nuclear family system) [11]. Due to the fact that numerous family members reside in one huge home, the joint family structure is regarded as a powerful social institution in rural areas of Pakistan [26]. As a result, grandparents, parents, uncles, and aunts protect children from violent behavior. They experience fewer issues than nuclear family households because they monitor their children’s daily screen time on TV and mobile devices. Additionally, children from joint families are happier than those from nuclear households; in contrast, children from nuclear families showed more problems, such as loneliness, aggression, and behavioral problems [25,27]. To our knowledge, there is a lack of deep information on animation violence in different family structures (nuclear and joint families) with gender variations. Therefore, a systematic approach is required to examine the impact of animation violence on primary school students from both family systems.

### 2.3. Viewing Animation Duration on Students’ Aggression

Viewing animations for a longer time on a daily basis is more harmful than any other experience. Children spend more time watching violent, nonviolent, animated, comedic, and non-comedic animated movies. Both violent and nonviolent animations play different roles in child behavior, causing less time to be spent with peers and friends. Furthermore, previously published studies demonstrate that children who watched violent animated movies frequently were more likely to experience violence and social isolation than those who watched nonviolent animations [9,10,28]. It was also found that watching violent animations with non-comedic content is prone to more aggressive behavior than watching violent animations with comedic content [22]. Generally, if the animations are violent, the child prefers to watch the favorite scenes or characters repeatedly on a daily basis [22]. Children learn these actions and reactions, i.e., kicking, hitting, and beating from animations, relatively faster than adults, which can make them more aggressive and ruder [22].

### 2.4. CBT and CBACT

Watching violent animations is directly associated with aggressive and behavioral disruption and mobilizes children toward dangerous situations [9]. This condition alarms parents, teachers, researchers, and policymakers to control these problems in children. In order to improve the behavior of students, a smart strategy or intervention is required to reduce and/or control animated-driven anger, and aggression in students [6]. Therefore, several different types of interventions, including CBT, parent-management training (PMT), problem solving, and coping power program (CCP), etc., have been applied to control aggression and the mental disorders of students [5,6,7,8]. However, compared to other psychotherapies, CBT is considered more effective at dealing with negative thoughts and emotions and enhancing behavior in a variety of populations [6,7,29]. It originated from two main theoretical frameworks: social learning theory (SLT) and cognitive social learning theory (CSLT). These theories have significantly contributed to the development and understanding of CBT as a highly effective therapeutic approach. CBT suggests that many people consider a new target using satisfying therapy by setting a new stage, which then explores the connection between thoughts, emotions, and behavior [6]. In principle, CBT is incorporated by a model known as a cognitive model that is based on feelings and behavior stemming from thoughts but not from exterior stimuli [6,29]. It is a simple model that imagines a link between emotions and behavior and is affected by the events’ perceptions. Previous studies have shown that CBT focuses on counseling to determine anger, aggression, and violent behavior in individuals by altering their thinking, emotion, and behavior performance [6,9,30]. Generally, it is a school-based strategy, but parental contribution is also important for the success of therapy. For example, the parent’s permission, the child’s information before and after the therapy, and the development of the environment during sessions of CBT skills are important [7]. Nonetheless, different kinds of CBT are used to emphasize one of these problems: (i) regulation of extreme anger; (ii) learning social problem-solving approaches; and/or (iii) developing social skills alternatives in children [31]. Furthermore, several pieces of supporting information on cognitive behavioral interventions (CBIs) can be obtained from the work presented by Barnes et al. [32], in which authors reviewed and provided a meta-analysis by explaining the potential role of school-based CBIs for the treatment of aggression in school-age children and adults in the United States.

CBACT is a kind of CBT and is a ten-session-based intervention generally designed to control or reduce anger and aggression by applying its three main mechanisms/components. These mechanisms are (i) arousal management, which recognizes the anger’s trigger as arousal management; (ii) cognitive restructuring, which supports modulating thinking; and prosocial skills therapy to provide alternate responses; and (iii) modeling and role-playing appropriate behavior [33,34,35]. These three components are based on skills that are developed during teaching ten sessions categorized into three blocks [35]. CBACT is applied to all kinds of participants, such as children, adolescents, and the elderly [34]. Previously published studies have shown that CBACT programs significantly reduce the aggression and aggressive behavior of primary school students [35]. However, these studies suggested that CBACT programs can be improved and assessed by providing homework to primary school students and involving parents to investigate children’s behavior in CBACT treatment and non-treatment (control) groups [35]. Hence, in this study, we applied the CBACT program to control and reduce the four dimensions of aggression (BPAQ) and three dimensions of behavior (CBQ-parent version) in animation-driven primary school students.

#### 2.4.1. Aims of the Present Study

This work proposes two kinds of studies (*Study I* and *Study II*). In *Study I*, the influence of violent and nonviolent animated movies on four dimensions of the BPAQ (anger (AN), physical aggression (PA), verbal aggression (VA), and hostility (HS)) and three dimensions of the CBQ (effort control (EC), surgency, and negative affectivity (NA)) of primary school students. Following that, we applied a CBACT program (*Study II*) to control and reduce aggressive behavior in primary school students selected from *Study I*.

Therefore, the specific objectives of the present study are to examine the influence of animation type (violent and nonviolent) and its in-depth effect on primary school students of different genders (male and female), family systems (nuclear and joint) with gender differences, viewing screen time duration (10 min, 20 min, and 30 min), and their control. This study provides new insights into educational and social psychology as well as the behavioral management of students.

#### 2.4.2. Research Hypotheses

The hypotheses formulated for *Studies I and II* are as follows:

i. There is a considerable influence of violent animations on the aggression of students compared to nonviolent animations.
ii. Male students are significantly affected by violent animation.
iii. Boys from nuclear families behave more aggressively than boys from the joint system after watching violent animation.
iv. There is a substantial influence of time spent watching animations on students’ aggression.
v. CBACT enhances students’ behavior performance while reducing or controlling aggression in them.

## 3. Study I: Effects of Animated Movies on Aggression and Behavior Performance of Primary School Students

### 3.1. Method

#### 3.1.1. Participants and Their Selection

**Figure 1:**
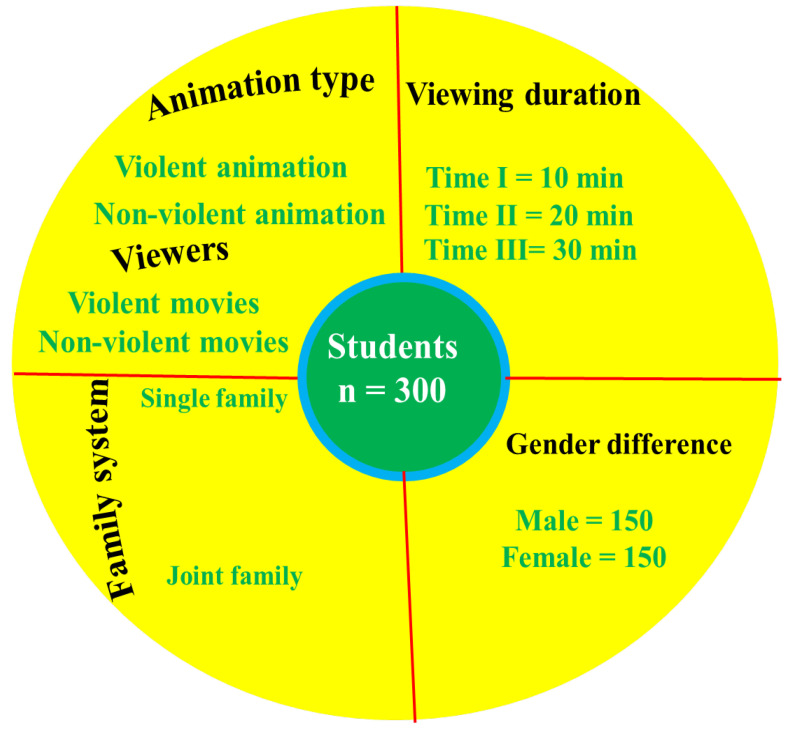
Schematic diagram illustrating the methodological framework of *Study I*.

*Study I* was approved by the experts of the authors’ department and the Ethics Research Committee of Soochow University. The sample of the study was 300 primary school students, and their parents were recruited from ten different schools in Pakistan. The students were selected with the simple random method with the help of class teachers; 150 students were male (50%) and the other 50% were female (Figure 1). Thirty students aged 7 to 9 years from grade 1 to grade 3 were selected from each school. All of the students were in good physical and mental condition. Parents were asked to parent–teacher meetings by their children on behalf of the class teachers. In front of the parents, the researcher thoroughly described the animation viewing activity and asked for their approval for the children to participate in this study as well as their own participation to complete the CBQ questionnaire. Out of 300 students, 50% were from joint families (boys ꞊ 62, girls ꞊ 88) and the other 50% were from nuclear families (boys ꞊ 71, girls ꞊ 79). For viewing animations, 50% of participants were randomly assigned to watch violent animated movies, and the other 50% were assigned to watch nonviolent animations. Children and their parents were administered the questionnaires before and after viewing violent and nonviolent animations.

#### 3.1.2. Overall Design

The overall study design was a 2 (type of animation watched: violent vs. nonviolent) × 2 (sex: male vs. female) × 2 (family system: nuclear vs. joint) × 2 (time duration: Time 1 vs. Time 2 vs. Time 3). The independent variables of the present study were animations, family systems, and viewing time duration, while aggression and behavioral performance were the dependent variables. The present study aimed to test the aforementioned hypotheses by investigating the influence of these independent variables on dependent variables.

#### 3.1.3. Measures

Two types of tools were used in the present study to examine the aggression and behavior performance of the students.

##### Aggression Questionnaire

The Buss–Perry Aggression Questionnaire (BPAQ) was used to observe aggression in children by following a review of the literature and to obtain the required data [36]. The BPAQ, consisting of 29 items, was previously used with primary school students to test student aggression [37,38]. The aggression of students was measured by using a 5-point Likert scale, from 1 ꞊ extremely uncharacteristic to 5 (extremely characteristic). Four well-defined aspects of the BPAQ, i.e., PA (9 items), VA (5 items), anger (An, 7 items), and hostility (HS, 8 items), were examined in this study. The Cronbach’s alpha coefficient, α (reliability), for the overall scale, is 0.89, while the scores for the four different facets are (i) PA ꞊ 0.85, (ii) VA ꞊ 0.72, (iii) AN ꞊ 0.83, and (iv) HS ꞊ 0.77. The questionnaire was shown to bilingual experts to obtain help with the questions in the students’ native language.

##### Behavior Questionnaire

The behavior performance of students was examined by using the Child Behavior.

Questionnaire-Very Short Form-Parent Form (CBQ-VSF-PF), consisting of 36 items and rated on a 7-point scale from 1 ꞊ extremely untrue to 7 ꞊ extremely true [39]. The CBQ-VSF-PF was in the student’s native language and was obtained by requesting the original author (Sam Putnam). The native language version of the questionnaire was translated by Nadia Ijaz and Arifa Siddique and then was further adapted by Attiya Siraj. Three broad dimensions, effort control (EC), surgency, and negative affectivity (NA), of students, were analyzed from the CBQ-VSF-PF. The alpha coefficients of the questionnaire ranged from 0.62 to 0.78 for the three subscales, and the estimated value of α for EC ranged from 0.62 to 0.78; for surgency, the value was ranked from 0.70 to 0.76, and 0.66 to 0.70 for NA [39].

#### 3.1.4. Experiment and Procedure

Prior to starting the experiment, the researcher visited each school with a list of ten previously released animated films (including cartoons and video game movies). This list of animated movies was provided as a Supplementary Material (Table S1). These animated films were famous in Pakistan and interested the students. Twenty parents were given the task of choosing one violent animation and an equal number of nonviolent animations (Table S1). The most voted animated films that were both violent (3 Bahadur) and nonviolent (Abdul Bari) were then chosen for the pilot and main studies. Before the experimental day, the CBQ was enclosed in the envelope, handed over to students, and students were asked to provide the questionnaire to their respective parents and bring it in the same manner as that delivered to them. The BPAQ was provided to the students, explained in the student’s native language, and then guided to mark in the appropriate box. The participants were gathered in separate rooms within the school to view animations. Both violent and nonviolent animations were shown to the participants. Additionally, each participant watched animated movies of either violent or nonviolent animations for three different durations: (i) Time 1 = 10 min; (ii) Time 2 = 20 min; and (iii) Time 3 = 30 min (Figure 1). After watching the animations three times (Time 1, Time 2, and Time 3), the toy-selecting task was conducted for violent and nonviolent viewers. After each viewing session, the students were presented with 60 toys—30 violent toys (of the same or a different type) and an equal number of nonviolent toys. These toys were bought at a nearby market or shop. These toys also included both violent and nonviolent animated film characters in some of their designs [10]. Finally, toys for violent and nonviolent animation were available for viewers to select. At the end of the animation viewing activity (Time 3), BPAQ was given to the students and they were instructed to mark the relevant box. The CBQ was placed in the envelope, and the students were advised to deliver it to their parents so they could complete it and return it to the researcher within two days in the way specified.

#### 3.1.5. Data Collection and Statistical Analysis

The data were collected through personal visits to each school and were analyzed by SPSS statistics software, version 21, and then ANOVA, Student’s *t* test, and the chi-square test were performed.

### 3.2. Results

#### 3.2.1. Main Effects of Animation Violence on Aggression and Behavior of Students

The *t* test method was applied to analyze the significant difference in aggression and behavior of the participants before and after viewing violent and nonviolent animations (Table 1). Our results are highly significant and showed that both aggression (four dimensions) and three dimensions of behavior largely increased after viewing violent animations, while they were slightly increased by watching nonviolent animations.

#### 3.2.2. Relationship between Animation, Gender, and Family System

Initially, we calculated the differences in the dimensions (DIF_dimension) of aggression and behavior of participants before and after watching the animation. For multivariate variance, the DIF_dimension was used as a dependent variable, while the animation type (violent, nonviolent), gender (male, female), and family system (single or nuclear, joint) with gender differences were studied as independent variables. The data showed that, with the exception of NA (from the behavior dimension), animation type, gender, and family system interacted strongly during each dimension of aggression and behavior (Table 2).

To analyze the in-depth relationship between animation gender, a two-by-two analysis was performed. Here, we used gender and animation type as independent variables and DIF as a dependent variable to run the multivariate analysis of variance (presented in Table 3). The results showed that the main effect of gender difference was significant in all dimensions of aggression and behavior, with the exception of HS (Table 3). The details of the DIF dimension versus violent and nonviolent animations are shown in Figure S1. It can be noted from the figure that violent animation viewers showed more aggression than nonviolent animation viewers. Figure S1 also shows that male viewers were more influenced by violent animations than female viewers, while female viewers had higher scores for nonviolent animations than male viewers.

Similar to aggression, the interaction between gender and animation type was significant during all the behavioral components, with the exception of surgency (Table 3). As reported in Figure S2, there was no substantial difference in increasing NA dimensions in boys and girls while viewing violent animations. After watching nonviolent animations, the NA dimension in boys was significantly lower than that in girls, while the EC value was lower in both genders (Figure S2). Multivariate analysis of variance of 2 (family system: joint, nuclear) 2 (gender: male and female) was carried out on differences in subjects’ scores (pre- and post-experiment, DIF) for each dimension, as presented in Table 4. It was observed that animations had a significant impact on both families, and all aggression dimensions (AN, PA, VA, and HS) were affected considerably by the viewing of violent animations, suggesting the negative role of animations in both family systems. However, the effect was different due to gender differences in these family systems. Thus, the DIF values of all dimensions for male students from nuclear families were higher, suggesting the substantial role of violent animations on the aggression of boys from nuclear families (Figure S3). Females from joint families remained dominant over nuclear family females and joint families’ boys. Similar to aggression, all the dimensions of behavior were significant (Table 4). For nuclear families, the alteration in behavior dimensions of male students was higher (except EC) than that of female students, while in joint families, female scores were dominant over male students (Figure S4). These findings showed that after viewing violent animations, male students from nuclear families and females from joint families were more prone to aggression and behavioral problems.

Multivariate analysis of variance of 2 (family system: joint, nuclear) and 2 (animation: violent, nonviolent) was carried out on DIF for each dimension of aggression and behavior (Table 5). It can be noted from the table that a significant interaction was associated between family system and animation in all dimensions of aggression (except AN and HS) and behavior (except EC).

#### 3.2.3. Impact of Animation Viewing Duration on Students

**Figure 2:**
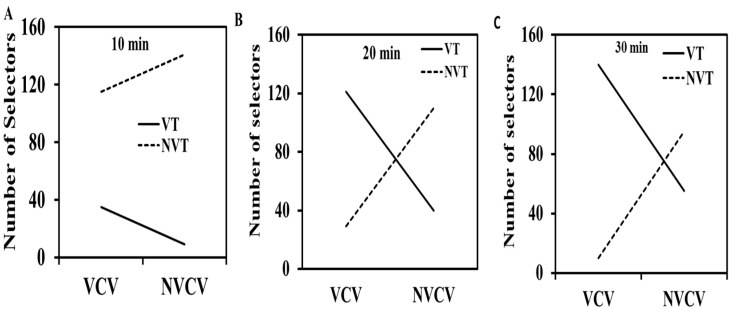
Impact of animation viewing duration on the aggression of students. The viewing time duration for 10, 20, and 30 min was displayed in (**A**–**C**).

Finally, we examined the effect of viewing screen time duration of animations on students using violent toys (VT) and nonviolent toys (NVT) (Figure 2 and Table 6). The table shows that all the scores of students were strongly significant. The toy activity for violent animation viewers (VCVs) and nonviolent animation viewers (NVCVs) was performed for a fixed duration of time (10–30 min) [10]. Furthermore, the chi-square results showed that the selection of VT increased with increasing viewing time duration (10–30 min) (Table 6). However, after 10 min of watching animations, the number of NVT selectors was significantly higher than that of VT selectors (Figure 2). Furthermore, after 20 min of viewing animations, the VT pickers were exceeded over NVT. The trend was even more pronounced for VT pickers after 30 min of viewing animations. Figure 2 also supports the Table 6 score, indicating that the aggressive problems of the students increased with increasing viewing time duration. These results were also attributed to the direct impact of animation violence on students’ aggression [10].

### 3.3. Discussion

#### 3.3.1. Violent Animations Significantly Increase Aggression and Behavior Performance

It was found in the present study that violent animations activate anger, which leads to aggression and behavioral problems in students and is supported by SLT, indicating that media, the environment, and peoples’ roles have influence over the child’s personality [3,4]. This alteration in children supported our first hypothesis. The findings are consistent with a study conducted by Habib and Soliman [10], which examined the impact of violent cartoons on children through a survey-experimental research design. The study [10] revealed that children who watched violent cartoons exhibited psychological and aggressive behavioral problems. Research from experts has suggested that when viewing violent animation content, aggressive and violent feelings can be increased in children, leading to aggressive behavior in children [12]. Other findings also supported our results that by viewing violent animated movies, short- and long-term aggressive and violent effects are raised in children [40]. Therefore, when some verbal, and physical aggression (fighting, hitting, and beating) scenes are shown in the animated movie, the child learns the actions and performs the same activities with their siblings and friends, even in non-dangerous contexts [40].

#### 3.3.2. Impact of Animations on Students with Gender Differences

It was observed that male students exhibited a higher level of aggression and behavioral problems compared to their female counterparts after viewing violent animated movies. The results provide support for the second hypothesis, which suggests that male students are significantly affected by violent animation. This finding in the present study indicates that there is a correlation between watching violent animated movies and the activation of aggressive thoughts and negative emotions in male students [22]. On the other hand, females may exhibit higher levels of self-control, enabling them to resist engaging in aggressive and violent situations [23,41]. Furthermore, compared to males, it was observed that the nonviolent score was higher in females. This finding suggests that nonviolent animated characters may have had a greater impact on female students, potentially leading to alterations in their behavior due to exposure to antisocial video content. It is possible that the repetitive and monotonous nature of such content bored female students, leading aggressive behavior in them. Our discussion is largely in support of previously reported studies, in which after viewing violent media, males showed more aggressive behavior than females [12,41].

#### 3.3.3. Impact of Animation Violence on Family Systems with Gender Differences

The influence of violent and nonviolent animations on the aggression and behavioral performance of students from different family systems with different genders was examined and compared. Two kinds of families are common in Pakistan: (i) nuclear or single-family systems and (ii) joint or extended family systems [42]. The findings of the study indicated that exposure to violence in animated content had a significant impact on both nuclear and joint family setups. However, there was a considerable difference in the scores based on gender, revealing that male students from nuclear families exhibited more aggressive and behavioral problems compared to those from joint families. This outcome supports the third hypothesis proposed in the study. The findings of this study are largely consistent with previously conducted research [43], which examined the emotional and behavioral issues experienced by primary school students. The study found that children from nuclear families tend to have fewer problems compared to those from joint families. Additionally, the results indicated that females from joint family systems exhibited relatively higher levels of aggression and behavioral problems compared to their counterparts from nuclear families. This behavior can be attributed to the fact that boys in joint families may have more opportunities for self-control while consuming violent content in movies, as they often watch them with larger family members. This suggests that males in joint families tend to participate more in indoor activities such as playing indoor games, watching television, or playing video games.

#### 3.3.4. Impact of Viewing Time Duration on the Aggression of Students

It was noted that the aggression of the students increased with increasing screen time duration of animation violence. This behavior suggests that even after viewing animated movies for a shorter period of time, students’ problems related to aggression arose [44]. These results supported our fourth hypothesis and are largely consistent with a previously reported study [9,40], which discussed that spending longer time duration on animated movies caused aggressive and behavioral disorders in viewers. These observations indicated that aggressive and behavioral problems increased with increasing viewing time duration (10 to 30 min), suggesting alterations in the feelings and thoughts of the students [10]. This situation can be harmful due to increasing the presence of T.V. in students’ bedrooms, also providing opportunities to access violent media content in the modern age via the internet, computers, and smartphones [44,45].

## 4. Study II: CBACT Reduces the Animation-Driven Aggressive and Behavioral Problems of Primary School Students

The purpose of *Study II* is to investigate the effectiveness of the CBACT program on primary school students selected from *Study I*. It is hypothesized that CBACT controls or reduces students’ aggression and improves their behavior performance.

### 4.1. Method

#### 4.1.1. Participants

The methodology of the present study was carried out by following a previously reported study with some modifications performed by Sukhodolsky et al. [35]. The study was approved by the experts of the author’s department and the Ethics Research Committee of their department (Soochow University). Initially, 50 students were selected on the basis of the findings of *Study I*. These students were chosen based on the results of Study I, which indicated that several students experienced a high level of anger, aggression, and behavioral issues after watching a violent animation. The selection process involved obtaining the consent of both the student’s parents and the willingness of their class teachers. For this purpose, the class teacher invited the parents of 50 students for parent–teacher meetings, but due to the unavailability of 4 parents, 4 students were excluded from the study. Therefore, CBACT was performed on 46 primary school students (masked for review) who were from six different schools and were studying in grade 1 to grade 3 (age = 7 to 9 years). The study was conducted in each school building, and out of 46 students, 23 (50%) were included in the CBACT treatment group, and the remaining 23 were included in the control group. This study was performed on a total of 12 groups (total = 46 students) of students (six groups for the control group and six for the CBACT group). Five student groups (for example, for the control group = 20 students) from each group consisted of four students, while the last group (sixth group) consisted of three students each. The parents of both student groups were invited for consent to guide them in the Child Behavior Questionnaire (CBQ) before and after the completion of the CBACT program. 

#### 4.1.2. Measures

As in *Study I*, two types of questionnaires were used as tools and completed by all students. These measures were used to examine the influence of CBACT on the aggression and behavior of animation-affected primary school students. A short form Buss–Perry Aggression Questionnaire (BPAQ-SF) was used to test the aggression of students, consisting of 12 items, followed by a literature review, and was used to obtain the necessary data [36,46,47].

The behavior and temperament of the students were assessed by the CBQ-VSF-PF that was purported to parents before and after the CBTACT program [39]. The items of the measure were rated on a 7-point Likert scale from 1 ꞊ extremely untrue to 7 = extremely true).

#### 4.1.3. Procedure

**Figure 3:**
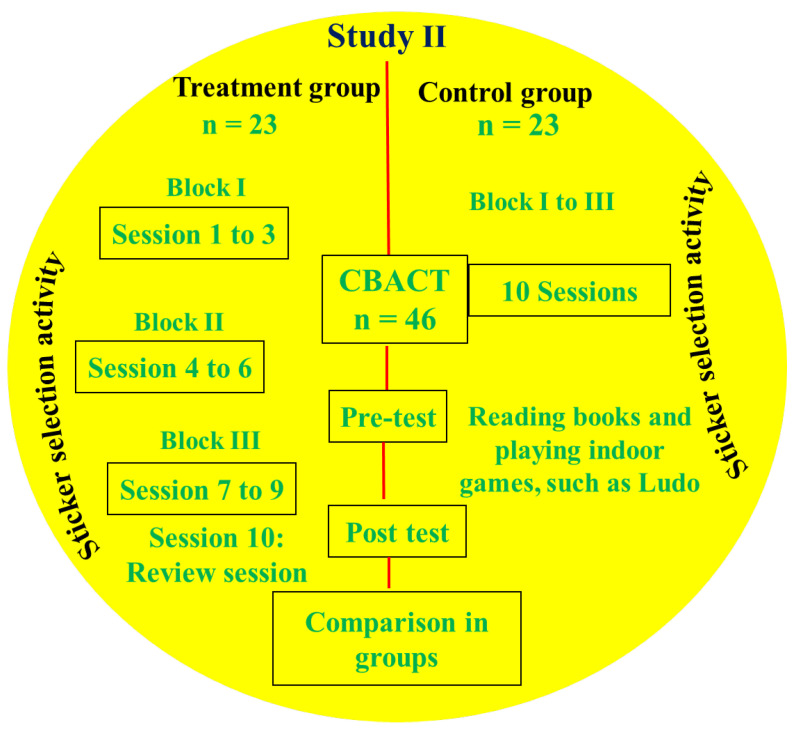
Schematic diagram showing the summary of the CBACT program (*Study II*).

CBACT was organized into three blocks with ten sessions, consisting of three sessions in each block (summarized in Figure 3), while the last session (session No. 10) was utilized to review the CBACT program. Students received 40 min of training during each session from the authors’ structured manual, which was followed and adapted from a previously reported study [35]. Students in the treatment group had received 40 min of training lessons for five weeks, and no more than two sessions were carried out in a week. The control group was engaged in games during the sessions. Each group had a leader (senior student), and his/her role was to retain discipline and control inappropriate circumstances, such as abuse, misbehavior, hitting, and fighting. The BPAQ was provided to students in both groups before starting activities in the control and treatment groups. The CBQ-VSF-PF was enclosed in the envelope and given to the students by advising them to hand it over to their respective parents for marking in boxes against each statement. It was also advised to students to bring the marked questionnaire in the same way that the researcher delivered it to them. The researcher motivated the students about the importance of attendance and advised them to attend all sessions of CBACT training. The student attendance authority was provided to the leader of each group to record the presence of the students every day [35]. It was also directed to the students to attend more than 70% of the training sessions (>7 sessions). However, the researcher briefly explained the missed sessions for understanding the students’ lessons, who missed the session. The class teachers also advised students to listen to the researcher and group leader carefully and to understand each other and the group leaders. Before starting the CBACT program, both groups conducted the sticker selection activity (Activity No. 1).

##### CBACT Treatment Group

This group consisted of 23 students, four students in five groups, and three in the sixth group. The treatment group was structured into blocks, with three sessions in each block, and the last (10th) session was used as a review session to obtain feedback about the impact of the CBACT program on students (Figure 3). At the end of each block, violent and nonviolent sticker selection activity was conducted. The detailed procedure of all CBACT sessions and their roles are described below.

First block: The first block of CBACT consisted of three sessions. The objective of the first session was to create a researcher–student relationship in a friendly environment and then to introduce the CBACT program and its importance. Furthermore, the researcher’s and group leader’s roles were briefly described during the first session. During the second session, the introduction of anger and aggression, their causes, and their impact on the students’ behavior were explained briefly. Different kinds of emotions and their expressions, such as anger, aggression, disgust, happiness, sadness, etc., were introduced, and their expressions were shown to students. As these expressions were easily seen in the animation movies, their relations with anger and aggression were addressed, and then the positive and negative impact of animations on them were discussed during the third session of the first block. For this purpose, a 10-min animation movie with violent content was shown to students, their body expression was noted, and then these changes were discussed with the students. With the completion of the first block, a homework assignment was given to students to remember the expressions of anger, aggression, and happiness and think about them while watching animations at home. After that, both the treatment and control groups performed the sticker selection activity (activity No. 2).

Second block: After receiving feedback from the first block, the second block was introduced, which consisted of the next three sessions (sessions 4 to 6). During session 4, animation-driven problems, such as negative feelings and anger in children and their role leading to aggression, violence, and behavioral problems, were further addressed. During session 5, some general questions were asked to students in a responsive environment to check their experience during a recent animation viewing activity: (i) Have you watched animations this week? (ii) Did the animations consist of violent content? (iii) What happened to you when you watched animations with violent content? (iv) Did the animations made you more aggressive and angry? In that polite environment, some students shared their experiences by showing and repeating actions from favorite animations and misbehaving with other students. After obtaining the student’s responses, the researcher addressed how to control the aggressive situations of students. The researcher explained that when you face such circumstances, try to control yourself. If it is difficult to control, start counting from 1 to 10 and breathe deeply at the time of aggression. Additionally, they also tried to stop watching violent animations by replacing them with nonviolent animations, i.e., moral, Islamic, comedic, and funny characters. During the start of the next (6th) session, the difference between animations and the real world was addressed comprehensively. For this difference, a short, animated movie was shown to them that had beautiful, animated worlds but also had a violent content which cannot exist in the real world. After watching the movie, the researcher further explained that while watching the violent animations, if anyone of you feels anger and aggression, then stop watching the animations, and think once in the real world: why am I aggressive? As I am not an animator, I am a real human. The animated character can make me more fear-child, what should I do in this situation? Remember the difference between animated animations and real-life world and act like a real-world human. At the end of the second block, homework was assigned to remember the difference between animation characters and real life and how to control their aggressive feelings and thoughts during and after watching violent activity animations. Before starting the third block sessions, both groups performed the sticker selection according to their own choice.

Third block: The third block dealt with techniques concerning physical movements and actions, which can further reduce the intensity and consequences of aggression by changing the body position of students. It was taught to students (session number 7) that if you have aggressive feelings make a practice of sitting stand or moving away from the action scene or refreshing with drinking water and washing your face or going away. Additionally, they should start walking and talking to themselves in a positive way in the real world and forget the violent animation content or play outdoor games. During the next session (session number 8) of CBACT, the awareness of positive feelings was inducted in participants by displaying a short moral movie to ensure their personal security to recognize the gravity of the occasion. Students were also advised to be ready and try to protect themselves and others from the physical and social consequences of anger and aggression. During the second-last session (session number 9), the researcher further addressed the generation of some positive feelings in their real life, which may develop confidence in students and overcome the violent animations’ influence on them. Additionally, this was taught to the students by following the lessons, and some positive changes in one’s mind can be acquired, such as am I safe? Can I hurt myself or anyone else if I get angry while watching violent animation and other violent situations? Will it disgrace me morally and socially if I just express my anger violently? and so on. In this way, the anger, aggression, and violence of the students were controlled effectively.

Similar to the first two blocks, a sticker selection activity (Activity No. 4) was performed after the end of the third block of CBACT, and students selected the sticker according to their own choice. To remember the whole training lesson, a review of all sessions was carried out during the last session (10th session) of the CBACT program. It guided students to watch animations during the weekend, and if the animation or any characters of the animation had a violent character, then CBACT strategies were applied in real life to overcome the incidences of aggression and violence.

##### Control Group

During all sessions of the three blocks of CBACT, the students from the control group were allowed to play some games, such as Ludo, by following the instructions of the researcher and group leader to retain discipline throughout the program. The control group had not received any specific training during these sessions. However, the group remained busy in the school building and enjoyed various games during the session time. Moreover, the students in the control group were under observation by group leaders during all activities of the treatment group. Similar to the treatment group, the sticker selection activity was also conducted for the control group just after the completion of each CBACT block.

At the end of CBACT, the BPAQ was provided to both groups of students to mark their own choices in the respective box. The CBQ-VSF-PF for parents was enclosed in the envelope and students were guided to provide it to their respective parents and returned it in the same way as they handed it over to them. A lunch party was arranged by the researcher for the teaching staff of the school. Additionally, the group leader and students (who made changes in their behavior at school and home) were gifted some money and other presentations.

### 4.2. Results

#### 4.2.1. Overall Aggression of Students

The overall values of the mean and standard deviation of BPAQ with respect to the *t* test before and after the CBACT program are reported in Table 7. The results (see Table 7) show that, with the exception of item No. 12 of the BPAQ-SF (*t* value ꞊1.955, *p* > 0.05), all the other items have significantly different values (*p* < 0.05) and higher *t* test scores.

#### 4.2.2. Evolution of Aggression and Behavior of Students

**Figure 4:**
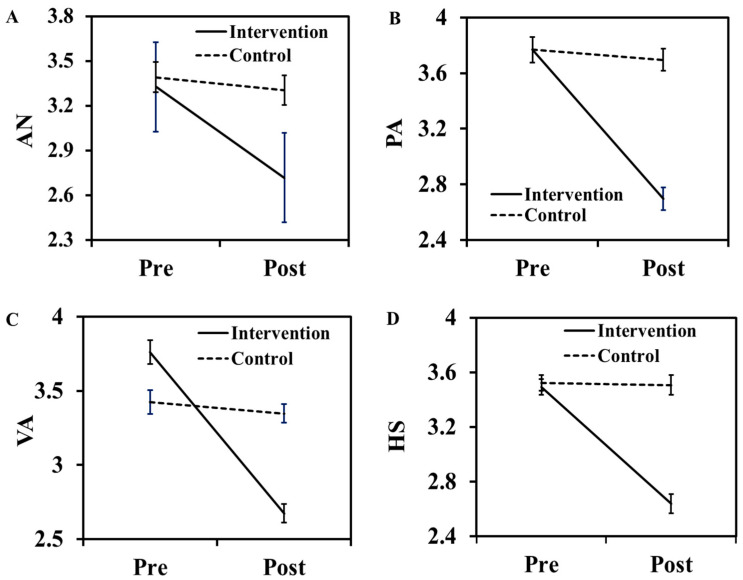
Evolution of students’ aggression before and after the CBACT program. Here (**A**–**D**) depicted anger (AN), physical aggression (PA), verbal aggression (VA), and hostility (HS).

**Figure 5:**
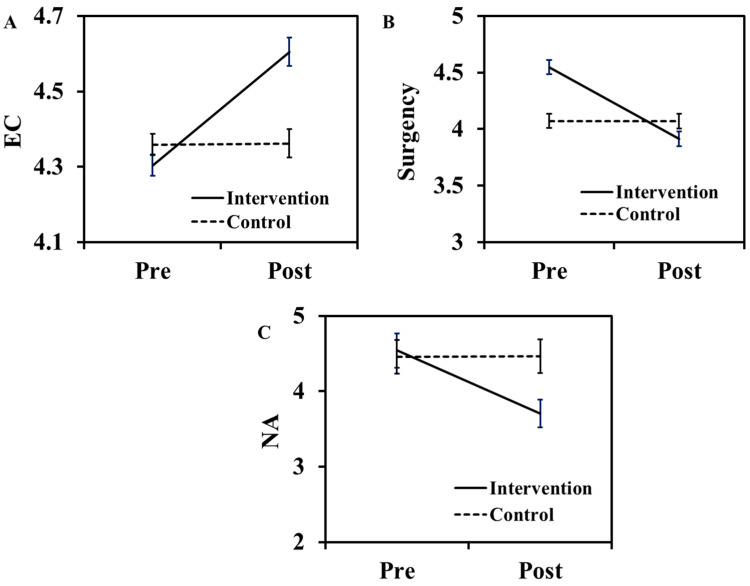
Changes in the behavioral dimensions of the students from the treatment and control groups were observed before and after CBACT. The (**A**–**C**) represent effort control (EC), surgency, and negative affectivity (NA) respectively.

The evolution of aggression and behavioral performance before and after CBACT is displayed in Figure 4A–D. The figure shows that all the dimensions of aggression (AN, PA, VA, and HS) were reduced by running the CBACT in the treatment group (Figure 4A–D). Similar findings were demonstrated in Figure 5A–C, in which the behavioral changes (surgency and NA) also declined with treatment in students, while the opposite behavior of CBACT was noted for the EC dimension (Figure 5A). The results related to the effect of CBACT for repeated measures analysis of variance on students’ aggression and behavior are depicted in Table 8 and Table 9. The intervention effect on Eta at a fixed differential value (df ꞊ 1) shows that, with the exception of VA, all dimensions of aggression related to Eta are significantly different (*p* < 0.05) (Table 8). 

Furthermore, Table 8 also displays that the Eta value in the CBACT group in all BPAQ dimensions was higher for VA and lower for AN. For behavioral problems, the NA had a higher Eta value, while a lower value of Eta was found in the EC (Table 9). A higher Eta score suggests a stronger response of the CBACT to the behavior of the students. The results of the repeated measures analysis (df ꞊ 1) of both aggression and behavioral problems in the intervention group were strongly significant (Table 8 and Table 9). The outcome of the sample effect analysis of aggression and behavioral dimensions is displayed in Table 10 and Table 11. The data show that the post-test scores of all aggression dimensions of CBACT students were relatively lower than those of the control group (Table 10). The *p*-value of the treatment group was highly significant (*p* < 0.0001), while the value was not significant in the control group (*p* > 0.05). The post-test scores of surgency and NA were also reduced but increased in the EC (Table 11). This behavior can be attributed to the strong impact of CBACT on surgency and NA due to the strengthening of bonding of children with parents and other family members [48]. However, a lower effect was seen for the EC dimension.

Finally, we ran the chi-square test at a constant *df* value (df ꞊ 1) by performing the sticker selection activity to examine the further role of the CBACT program on students (Table 12). The activity was performed four times on both groups, following the selection of violent (VS) and nonviolent stickers (NVS) by both groups. The results showed that prior to the CBACT program (Activity No. 1), a larger number of students (19 in the treatment group and 20 in the control group) picked the VS. However, after applying CBACT, the VS selection was reduced continuously from Activity No. 2 to Activity No. 4 (VS picker 15 to 1), while the numbers were increased in the control group (VS picker 19 to 21).

### 4.3. Discussion

In this study, we sought to determine the influence of CBACT in reducing the animation-driven anger, aggression, and aggressive behavior of primary school students. The CBACT program is a CBT-based training with the support of aggression theories, especially SLT, which can be helpful in understanding behavioral problems as well as reducing media-driven anger and aggression in students [48,49]. Anger is a complex emotion that is often associated with the “fight or flight” response. In the context of primary school students, CBACT has been implemented in three blocks, each consisting of ten sessions [35,48]. Although CBACT was conducted as a school-based intervention, the researchers also sought to involve parents in the intervention process at home. Consistent with previously reported studies, the treatment group exhibited low aggression scores. This finding aligns with the results of previous research that has also shown low levels of aggression in treatment groups [35,50,51]. For example, a study conducted by researchers [51] examined the effects of self-awareness skills and anger management training on a sample of 395 male students from five middle schools. The authors of this study reported that both self-awareness skills and anger management training had a positive influence on reducing students’ aggression. More specifically, the post-test score showed that all dimensions of the BPAQ and two dimensions of the CBQ (surgency and NA) of the treatment group were reduced more than those of the control group. However, CBACT has been found to have a significant impact on students’ attitudes, specifically in helping them control animation-driven aggression and aggressive behavior [5,35,52]. The results of the current study are largely consistent with a previously reported study conducted by Mushtaq et al. [5] on primary school students in Pakistan. In their study, the authors implemented the Coping Power Program (CPP) to reduce anger, aggression, and improve behavioral performance among students. Similar to the findings of the current study, the CPP intervention was effective in controlling and reducing anger in boys, as well as enhancing positive behavior in students [5]. Moreover, the results related to repeated and sample effect analysis of the present study supported the above discussion, demonstrating that aggression and behavioral dimensions were substantially reduced, suggesting the effective role of CBACT over these dimensions. Moreover, it was noted that the temporary screen viewing of violent animations had led the students toward the dangerous situation, linked with anger, hostility, verbal, and physical aggression, as well as behavioral problems, but these issues can be reduced or handled by the CBACT program up to a certain limit [53]. Our discussion is largely in line with the study described by Cantor and Wilson [54], who reviewed the targeted role of media violence in adolescents’ aggression and its control by using interventions to reduce aggressive behavior. Furthermore, our finding was particularly effective for lower grade students. This aligns with a previously published study conducted by Sukhodolsky et al. [55], which specifically examined the impact of behavioral interventions in preventing aggression among children. The chi-square test results of both groups during violent (VS) and nonviolent sticker (NVS) selection indicated that with treatment, the number of NVS pickers was increased more than that of the control group, while the number of VS selectors (Activity No. 2 to Activity No. 4) was reduced in the treatment group. This behavior indicates the effective role of the CBACT program, which may have played a significant role in differentiating between the portrayed animated animation and the real human world. *Study I* showed the fast mimicking of aggression and aggressive behavior in primary school students; however, a rapid reduction in anger and aggressive behavioral problems by learning the CBACT program was also observed in the students. Additionally, CBACT improved the cognitive behavior and manner of students, and at the end of CBACT, fewer VS selectors were seen in the treatment group, which supports our fifth hypothesis. Another effective aspect of the present study was to engage the students in homework assignments at the end of each block. Since parents were also involved during the CBACT program, most of the treatment group’s students completed the homework at home. Our findings provide considerable support for social learning theory, indicating that school and home environments, especially parent–child bonds, play a significant role in the reduction of screen activity as well as the development of the child’s personality [3,4,56]. Nonetheless, the visual observations of group leaders and researchers during all the training sessions illustrated that CBACT was organized in a systematic way to reduce the aggressive and behavioral complications of primary school students. Furthermore, CBACT was run in a polite way; therefore, students followed it in a friendly environment. This approach resulted in the successful implementation of anger-control interventions. The findings indicate that after receiving the CBACT program, students did not completely abstain from watching violent movies. However, they demonstrated improved control over their aggression while viewing violent animations.

## 5. Limitations and Future Directions

*Study I* set out the effects of animation violence on the aggression and behavior problems of students, which can be explained on the basis of five theories of aggression^4^. However, our findings are interesting and can be useful in promoting social psychological behavior as well as the educational achievements of students. However, some limitations can be considered in *Study I*.

First, the sample of the study was from one district and focused only on one region of the country, and no variation in culture was demonstrated. Therefore, future studies should be conducted and compared with students from other regions of the country (province) or countries such as China. Second, the current study was restricted to primary school students only and must be conducted with students in higher grades in the future. Third, due to the limited communication, the parents showed hesitation to provide information about their respective children. Fourth, the viewing screen time duration was limited from 10 to 30 min and should be examined for a longer time in future studies. Fifth, the current study described the influence of violent animations on the aggression and behavioral problems of students, but how to tackle these issues was not investigated. Therefore, *Study II* was needed to overcome these problems by employing interventions on the aggressive behavior of students [35,57]. Some limitations can be considered in *Study II*, which are provided below:

First, CBACT was only performed on animation-driven aggressive and behavioral problems, and future studies should be conducted on other media, such as video games. 

Second, the fidelity of the CBACT program was not assessed through therapist or observer ratings, which is the limitation of the *Study II*. 

Third, gender differences and family systems were not considered in this study. If these factors are studied, more reliable results can be achieved. Fourth, the sample of the study was only selected from one part of the state of the country; therefore, future research should be assessed in other areas of the province or the country (China). Fifth, due to the low age and grades of students, the CBACT program has not eliminated the students from viewing violent animations entirely. Therefore, future studies can be developed on violent media and its control using other interventions, such as parent–child interaction therapy (PCIT), social activity therapy (SAT), and coping power program (CCP) over middle or high school students [5,57,58,59]. 

Despite the above limitations, our findings are significant for theoretical and experimental work, which can be useful in improving the social and moral behavior of students.

## 6. Conclusions

This work is the combination of two studies, addressing the impact of animation such as cartoons and video game movies on aggression and behavioral performance as well as their control in primary school students.

The results of *Study I* showed that violent animations played a significant role in the aggression and behavioral problems of male students compared to female students, while girls were more affected by viewing nonviolent animations. Moreover, males from nuclear families and females from joint families had more aggressive and behavioral problems, indicating that boys enjoyed groups with larger family members more. It was also demonstrated that the aggression in students increased with increasing screen time duration of animations (10 to 30 min), suggesting the alteration in feelings and thoughts of the students with watching time. The findings of the current study may be useful to improve the social psychological behavior and educational achievements of students.

In *Study II*, the CBACT program was used as a cognitive behavioral therapy to control all dimensions of aggression and behavioral problems. A significant improvement can be seen in the treatment group’s students after post-test analysis, which concludes that CBACT reduced the anger and aggression of the students by controlling the aggressive thoughts, feelings, and anger of the students. Moreover, it was also suggested that temporary screen viewing of violent animations caused anger in students, which led to aggression and aggressive behavior in students. However, due to many rehearsals of CBACT, these students’ problems were controlled. The results conclude that with CBACT treatment, the primary school students were not eliminated from viewing violent animations entirely, but their aggressive and behavioral issues were reduced while watching violent animations. Nevertheless, our findings provide significant awareness for parents to spend leisure time with their children and should limit their animation watching time by replacing them with outdoor games [60]. Moreover, future studies are needed to examine the influence of violent media (violent movies and/or video games) on aggression and behavioral problems and their control on higher-grade students.

## Figures and Tables

**Table 1:** Aggression and behavior of primary school students before and after watching violent and nonviolent animated movies.

Animation	*N*	Measure	Dimensions	Pre	Post	*t*	*p*
Violent	150	aggression	AN	1.37 (0.21)	2.82 (0.63)	−28.86	0.000
	150		PA	1.34 (0.24)	2.41 (0.75)	−18.55	0.000
	150		VA	1.37 (0.22)	2.19 (0.56)	−18.15	0.000
	150		HS	1.25 (0.24)	2.09 (0.49)	−20.15	0.000
	150	behavior	EC	4.66 (0.22)	4.36 (0.17)	16.39	0.000
	150		Surgency	3.72 (0.17)	4.23 (0.27)	−23.35	0.000
	150		NA	3.68 (0.18)	4.36 (0.21)	−33.04	0.000
Nonviolent	150	aggression	AN	1.38 (0.16)	1.67 (0.57)	−6.41	0.000
	150		PA	1.34 (0.21)	1.57 (0.5)	−5.97	0.000
	150		VA	1.37 (0.19)	1.55 (0.43)	−5.47	0.000
	150		HS	1.29 (0.2)	1.46 (0.37)	−5.78	0.000
	150	behavior	EC	4.7 (0.23)	4.61 (0.25)	7.30	0.000
	150		Surgency	3.6 (0.18)	3.64 (0.19)	−3.18	0.002
	150		NA	3.59 (0.18)	3.81 (0.35)	−8.59	0.000

Note: AN, PA, VA, HS, EC, and NA are abbreviated to anger, physical aggression, verbal aggression, hostility, effortful control, and negative affectivity.

**Table 2:** Relationship of DIF_dimension with animation, family system, and gender difference of students.

Dependent Variables	Interaction	*F*	*p*	*Eta*
DIF_AN	Family × Animation × Gender	19.046	0.000	0.061
DIF_PA	Family × Animation × Gender	25.701	0.000	0.081
DIF_VA	Family × Animation × Gender	14.591	0.000	0.048
DIF_HS	Family × Animation × Gender	12.440	0.000	0.041
DIF_EC	Family × Animation × Gender	7.235	0.008	0.024
DIF_Surgency	Family × Animation × Gender	13.725	0.000	0.045
DIF_NA	Family × Animation × Gender	0.302	0.583	0.001

**Table 3:** Relationship of DIF_dimension with animation and gender of students.

Dependent Variables	Independent Variables	*F*	*p*	*Eta*
DIF_AN	Gender	0.863	0.354	0.003
	Animation	146.359	0.000	0.498
	Gender × Animation	8.203	0.004	0.027
DIF_PA	Gender	0.060	0.806	0.000
	Animation	75.709	0.000	0.339
	Gender × Animation	13.115	0.000	0.043
DIF_VA	Gender	0.249	0.618	0.001
	Animation	68.573	0.000	0.317
	Gender × Animation	30.971	0.000	0.095
DIF_HS	Gender	24.117	0.000	0.076
	Animation	89.379	0.000	0.377
	Gender × Animation	0.280	0.597	0.001
DIF_EC	Gender	27.375	0.000	0.085
	Animation	51.198	0.000	0.258
	Gender × Animation	18.273	0.000	0.058
DIF_Surgency	Gender	0.300	0.585	0.001
	Animation	182.346	0.000	0.553
	Gender × Animation	0.637	0.425	0.002
DIF_NA	Gender	6.340	0.012	0.021
	Animation	105.845	0.000	0.418
	Gender × Animation	18.240	0.000	0.058

**Table 4:** Analysis of variance of DIF_dimension on gender difference and family system.

Dependent Variables	Independent Variables	*F*	*p*	*Eta*
DIF_AN	Gender	0.003	0.955	0.000
	Family	0.369	0.544	0.001
	Gender × Family	51.062	0.000	0.147
DIF_PA	Gender	1.041	0.309	0.004
	Family	2.344	0.127	0.008
	Gender × Family	63.778	0.000	0.177
DIF_VA	Gender	1.513	0.220	0.005
	Family	1.084	0.299	0.004
	Gender × Family	58.017	0.000	0.164
DIF_HS	Gender	11.910	0.001	0.039
	Family	0.431	0.512	0.001
	Gender × Family	36.367	0.000	0.109
DIF_EC	Gender	17.066	0.000	0.055
	Family	1.139	0.287	0.004
	Gender × Family	11.791	0.001	0.038
DIF_Surgency	Gender	0.696	0.405	0.002
	Family	0.715	0.398	0.002
	Gender × Family	20.604	0.000	0.065
DIF_NA	Gender	2.247	0.135	0.008
	Family	0.482	0.488	0.002
	Gender × Family	19.695	0.000	0.062

**Table 5:** Analysis of variance of DIF_dimension for animation and family system.

Dependent Variables	Independent Variables	*F*	*p*	*Eta*
DIF_AN	Family	0.796	0.373	0.003
	Animation	142.290	0.000	0.491
	Family × Animation	0.650	0.421	0.002
DIF_PA	Family	0.300	0.585	0.001
	Animation	73.760	0.000	0.333
	Family × Animation	4.121	0.043	0.014
DIF_VA	Family	0.004	0.952	0.000
	Animation	65.929	0.000	0.309
	Family × Animation	9.098	0.003	0.030
DIF_HS	Family	0.628	0.429	0.002
	Animation	84.344	0.000	0.364
	Family × Animation	1.821	0.178	0.006
DIF_EC	Family	0.004	0.952	0.000
	Animation	45.320	0.000	0.235
	Family × Animation	2.387	0.123	0.008
DIF_Surgency	Family	0.292	0.589	0.001
	Animation	177.160	0.000	0.546
	Family × Animation	4.929	0.027	0.016
DIF_NA	Family	0.382	0.537	0.001
	Animation	94.764	0.000	0.391
	Family × Animation	2.949	0.087	0.010

**Table 6:** Animations viewing duration and selection of violent (VT) and nonviolent (NVT) toys.

Duration	Viewer	Toy		*df*	(*ηp*^2^)	*X* ^2^	*F*	*p*
		VT	NVT					
10 min	VCV	35	115	1	0.24	18.004	9.5	0.000
	NVCV	9	141					
20 min	VCV	121	29	1	0.54	87.95	61.88	0.000
	NVCV	40	110					
30 min	VCV	140	10	1	0.59	105.86	81.68	0.000
	NVCV	55	95					

Note: VCV and NVC stand for violent cartoon viewers and nonviolent cartoon viewers, respectively.

**Table 7:** Results of *t* test by comparing the means of pre-and post-treatment students’ aggression.

Items	*N*	Pre	Post	*t*	*p*
		*M* (*SD*)	*M* (*SD*)		
agg1	46	3.7 (0.66)	2.93 (0.77)	7.012	0.000
agg2	46	3.52 (0.55)	2.91 (0.69)	5.334	0.000
agg3	46	3.76 (0.71)	2.93 (0.74)	6.389	0.000
agg4	46	3.87 (0.62)	3.02 (0.91)	6.263	0.000
agg5	46	3.98 (0.71)	3.43 (0.81)	6.298	0.000
agg6	46	3 (0.89)	2.78 (0.81)	2.876	0.006
agg7	46	3.91 (0.69)	3.35 (0.9)	5.327	0.000
agg8	46	3.63 (0.61)	3.13 (0.88)	6.191	0.000
agg9	46	3.37 (0.49)	3.17 (0.64)	2.446	0.018
agg10	46	3.63 (0.77)	3.22 (0.87)	5.182	0.000
agg11	46	3.74 (0.61)	3.3 (0.7)	5.056	0.000
agg12	46	2.8 (0.65)	2.67 (0.63)	1.955	0.057

**Table 8:** Outcome results of repeated measures analysis of variance on students’ aggression.

Dimensions	Effect	*df*	*F*	*p*	*Eta*
AN	intervention	1	50.968	0.000	0.537
	Group	1	6.092	0.018	0.122
	intervention × Group	1	28.670	0.000	0.395
PA	intervention	1	231.148	0.000	0.840
	Group	1	18.900	0.000	0.300
	intervention × Group	1	176.333	0.000	0.800
VA	intervention	1	237.621	0.000	0.844
	Group	1	3.352	0.074	0.071
	intervention × Group	1	179.508	0.000	0.803
HS	intervention	1	120.365	0.000	0.732
	Group	1	29.344	0.000	0.400
	intervention × Group	1	112.474	0.000	0.719

**Table 9:** Outcome results of repeated measures analysis of variance in students’ behavior.

Dimensions	Effect	*Df*	*F*	*p*	*Eta*
EC	intervention	1	49.468	0.000	0.529
	Group	1	5.101	0.029	0.104
	intervention × Group	1	47.141	0.000	0.517
Surgency	intervention	1	196.957	0.000	0.817
	Group	1	3.297	0.076	0.070
	intervention × Group	1	192.506	0.000	0.814
NA	intervention	1	360.772	0.000	0.891
	Group	1	33.633	0.000	0.433
	intervention × Group	1	373.542	0.000	0.895

**Table 10:** Outcome results of sample effect analysis on treatment and control groups for BPAQ.

Dimensions	Group	Pre	Post	*p*
AN	treatment	3.33 (0.1)	2.72 (0.1)	0.000
	control	3.39 (0.1)	3.3 (0.1)	0.214
PA	treatment	3.77 (0.55)	2.7 (0.46)	0.000
	control	3.77 (0.29)	3.7 (0.28)	0.180
VA	treatment	3.76 (0.08)	2.67 (0.06)	0.000
	control	3.42 (0.08)	3.35 (0.06)	0.161
HS	treatment	3.49 (0.06)	2.64 (0.07)	0.000
	control	3.52 (0.06)	3.51 (0.07)	0.797

**Table 11:** Outcome results of sample effect analysis on treatment and control groups for CBQ.

Dimensions	Group	Pre	Post	*p*
NE	intervention	4.3 (0.03)	4.61 (0.04)	0.000
	control	4.36 (0.03)	4.36 (0.04)	0.906
Surgency	intervention	4.55 (0.06)	3.91 (0.06)	0.000
	control	4.07 (0.06)	4.07 (0.06)	0.911
NE	intervention	4.54 (0.04)	3.71 (0.05)	0.000
	control	4.46 (0.04)	4.46 (0.05)	0.815

**Table 12:** Results of violent and nonviolent sticker selection by control and treatment groups.

Sticker Selection		Group		Chi-Square *df*		*p*
		Treatment	Control			
Activity 1	VS	19 (19)	20 (20)		1.000	1.000
	NVS	4 (4)	3 (3)			
Activity 2	VS	15 (15)	19 (17)	1.804	1.000	0.179
	NVS	8 (6)	4 (6)			
Activity 3	VS	6 (13.5)	21 (13.5)	20.175	1.000	0.000
	NVS	17 (9.5)	2 (9.5)			
Activity 4	VSNVS	1 (11) 22 (12)	21 (11) 2 (12)	34.848	1.000	0.000

Note: VS and NVS are abbreviated to violent sticker selectors and nonviolent sticker selectors.

## Data Availability

The data that support the results of the present study will be available from the corresponding authors upon request.

## Supplementary Materials

The following supporting information can be downloaded at: https://www.mdpi.com/article/10.3390/bs13080659/s1, Table S1: Mean and standard deviation of parents’ selection (n = 20) of violent and nonviolent animation from 10 different animated movies; Figure S1: Relationship between DIF_dimension and animation type on the aggression of students with gender differences. Here Figures A, B, C, and D displayed anger (AN), physical aggression (PA), verbal aggression (VA), and hostility (HS), respectively; Figure S2: Relationship between DIF dimension and animation type on the behavioral changes of students with gender differences. The Figures A, B, and C in the Figure S2 represent effort control (EC), surgency, and negative affectivity (NA), respectively; Figure S3: Impact of DIF_dimension on students’ aggression, showing (A) anger (AN), (B) physical aggression (PA), (C) verbal aggression (VA), and (D) hostility (HS), respectively. The students were from different family systems with gender differences.; Figure S4: Impact of DIF_dimension on behavior changes of students from different family systems with gender differences. Here Figures A, B, and C represented effort control (EC), surgency, and negative affectivity (NA), respectively.
