# Supplementary material for: Effects of Animated Movies on the Aggression and Behavior Performance of Primary School Students and Their Control Using a Cognitive Behavioral Anger-Control Training (CBACT) Program
Source: Behav Sci (Basel). 2023 Aug 6;13(8):659. doi: 10.3390/bs13080659 (PMC10451846; doi:10.3390/bs13080659)
Supplement: Supplementary file 1 [file behavsci-13-00659-s001.zip › behavsci-2452367-supplementary.pdf]

Supplementary Material

Effects of animated movies on the aggression and behavior performance of primary school students and their control using a cognitive behavioral anger-control training (CBACT) program

Table S1. Mean and standard deviation of parents' selection (n = 20) of violent and nonviolent animation from 10 different animated movies.

S. No.	Animation Name	*M*	*SD*
1	3 Bahadur	1.000	.000
2	Call of duty	1.100	.308
3	Counter strike	1.048	.224
4	Grand Theft Auto	1.155	.366
5	Subway Surfers	1.450	.510
6	Abdul Bari	2.000	.000
7	Candy Crush Tales	1.950	.224
8	Motu Patlu	1.254	.444
9	Burka Avenger	1.399	.503
10	Talking Tom	1.855	.366

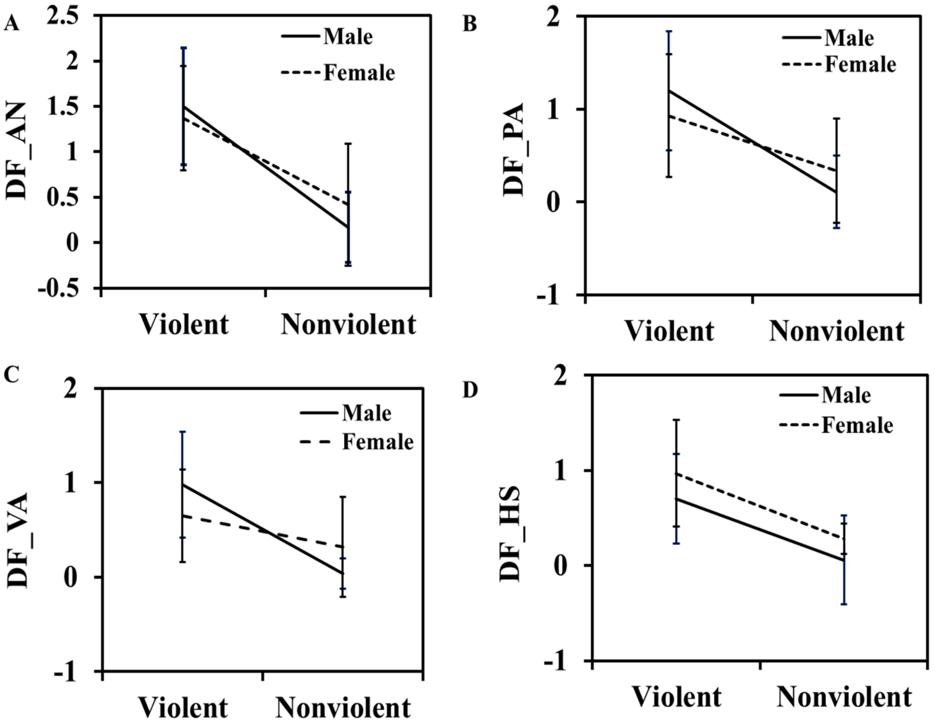

Figure S1. Relationship between DIF_dimension and animation type on the aggression of students with gender differences. Here Figures A, B, C, and D displayed anger (AN), physical aggression (PA), verbal aggression (VA), and hostility (HS), respectively

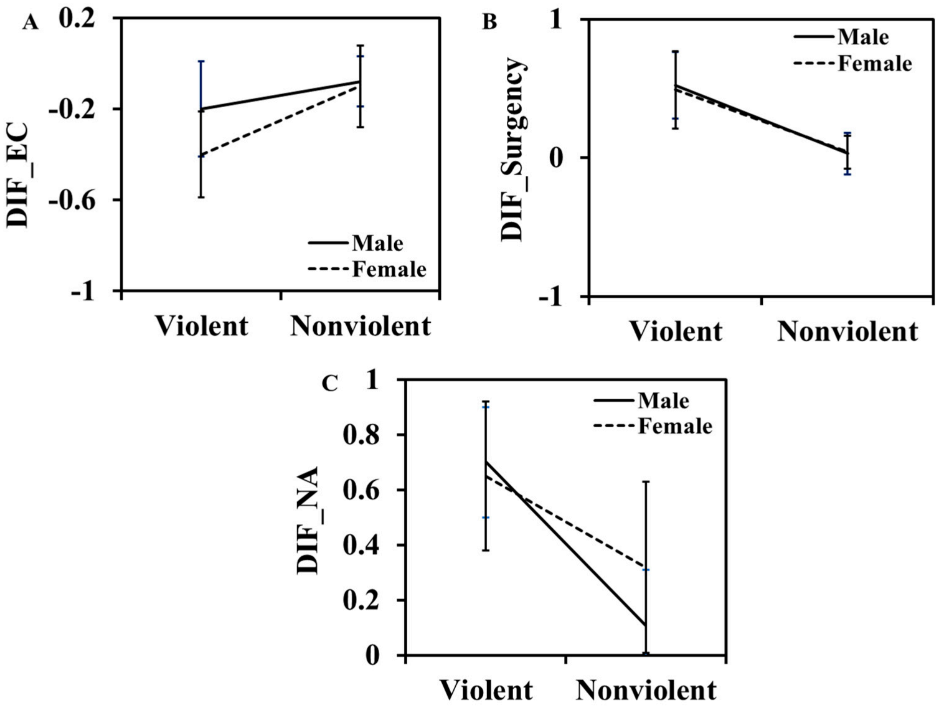

Figure S2. Relationship between DIF dimension and animation type on the behavioral changes of students with gender differences. The Figures A, B, and C in the Figure S2 represent effort control (EC), surgency, and negative affectivity (NA), respectively

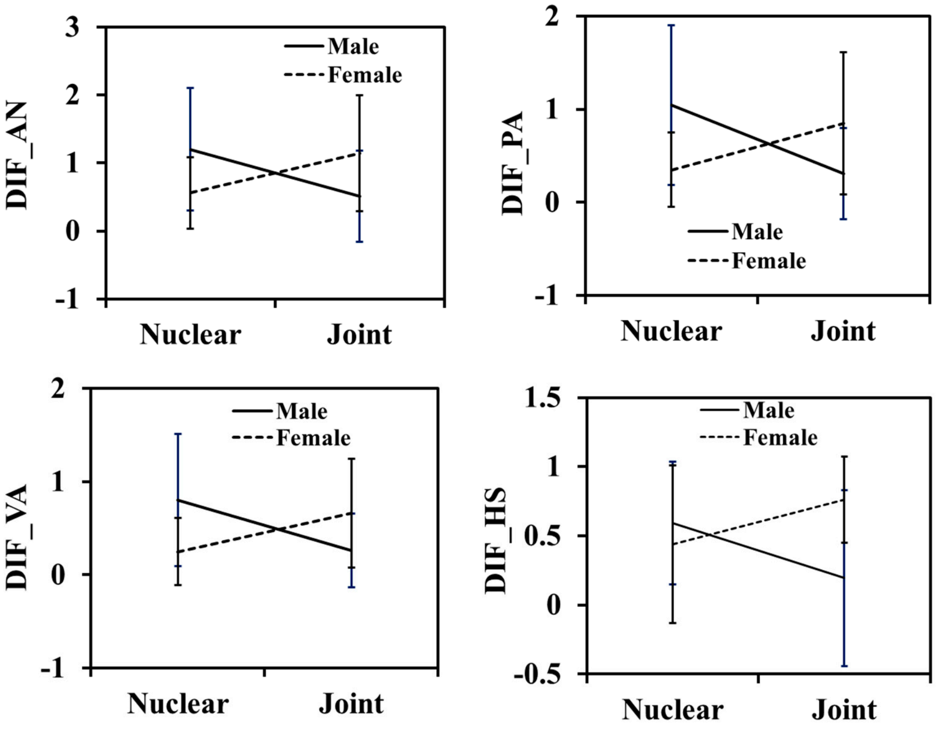

Figure S3. Impact of DIF_dimension on students' aggression, showing (A) anger (AN), (B) physical aggression (PA), (C) verbal aggression (VA), and (D) hostility (HS), respectively. The students were from different family systems with gender differences.

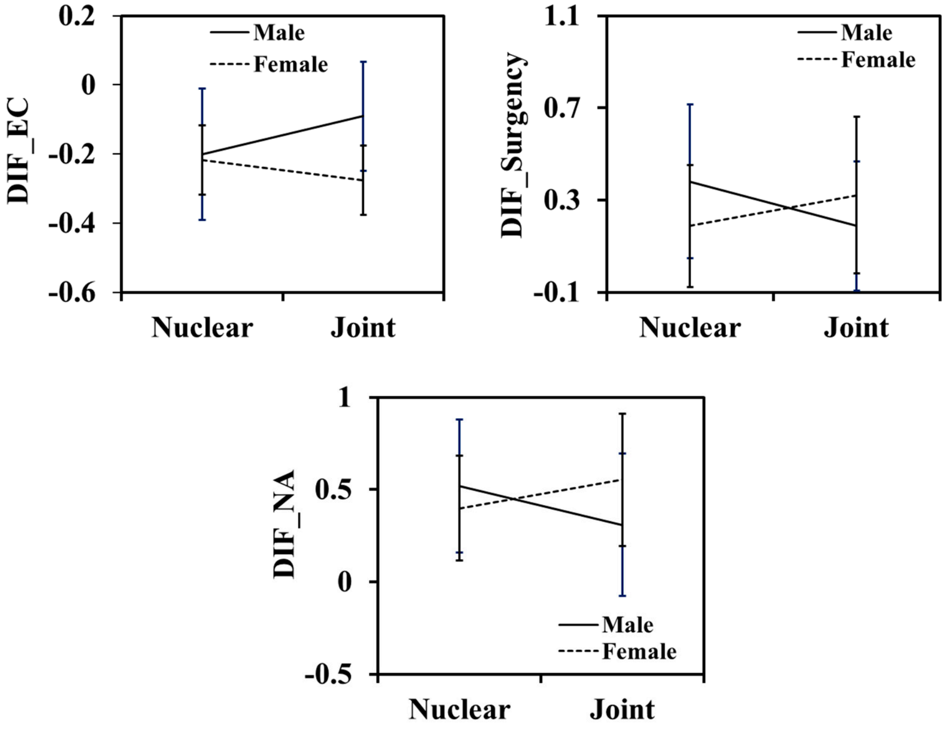

Figure S4. Impact of DIF_dimension on behavior changes of students from different family systems with gender differences. Here Figures A, B, and C represented effort control (EC), surgency, and negative affectivity (NA), respectively
